# Icariin-loaded hydrogel with concurrent chondrogenesis and anti-inflammatory properties for promoting cartilage regeneration in a large animal model

**DOI:** 10.3389/fcell.2022.1011260

**Published:** 2022-11-24

**Authors:** Songshan Xu, Shaohua Zhao, Yanpeng Jian, Xinwei Shao, Dandan Han, Fan Zhang, Chen Liang, Weijie Liu, Jun Fan, Zhikui Yang, Jinge Zhou, Wenqiang Zhang, Yigong Wang

**Affiliations:** ^1^ Department of Spinal Cord Surgery, Xuchang Central Hospital, Henan University of Science and Technology, Xuchang, China; ^2^ Medical Imaging Center, Xuchang Central Hospital, Henan University of Science and Technology, Xuchang, China; ^3^ Department of Nursing, Xuchang Central Hospital, Henan University of Science and Technology, Xuchang, China; ^4^ Department of Orthopaedics, The First Hospital of Shandong First Medical University and Shandong Provincial Qianfoshan Hospital, Jinan, China

**Keywords:** icariin, anti-inflammatory, chondrogenesis, large animal, hydrogel

## Abstract

Currently, an effective repair method that can promote satisfactory cartilage regeneration is unavailable for cartilage damages owing to inevitable inflammatory erosion. Cartilage tissue engineering has revealed considerable treatment options for cartilage damages. Icariin (ICA) is a flavonoid component of *Epimedii folium* with both chondrogenic and anti-inflammatory properties. In this study, we prepared an ICA/CTS hydrogel by loading ICA into chitosan (CTS) hydrogel to impart chondrogenesis and anti-inflammatory properties to the ICA/CTS hydrogel. *In vitro* results revealed that ICA showed sustained release kinetics from the ICA/CTS hydrogel. In addition, compared to the CTS hydrogel, the ICA/CTS hydrogel exhibited a favorable *in vitro* anti-inflammatory effect upon incubation with lipopolysaccharide pre-induced RAW264.7 macrophages, as indicated by the suppression of inflammatory-related cytokines (IL-6 and TNF-α). Additionally, when co-cultured with chondrocytes *in vitro*, the ICA/CTS hydrogel showed good cytocompatibility, accelerated chondrocyte proliferation, and enhanced chondrogenesis compared to the CTS hydrogel. Moreover, the *in vitro* engineered cartilage from the chondrocyte-loaded ICA/CTS hydrogel achieved stable cartilage regeneration when subcutaneously implanted in a goat model. Finally, the addition of ICA endowed the ICA/CTS hydrogel with a potent anti-inflammatory effect compared to what was observed in the CTS hydrogel, as confirmed by the attenuated IL-1β, IL-6, TNF-α, and TUNEL expression. The prepared ICA/CTS hydrogel offered an effective method of delivery for chondrogenic and anti-inflammatory agents and served as a useful platform for cartilage regeneration in an immunocompetent large animal model.

## Introduction

Cartilage damage is a common clinical condition that triggers pain and dysfunction in cartilaginous tissues. It frequently occurs owing to congenital and/or acquired conditions, such as trauma, tumor, and inflammation (Krishnan and Grodzinsky, 2018). Treating this clinical condition is challenging because of its poor capacity for self-repair owing to the lack of involvement of the vascular, nervous, and lymphatic systems, which causes poor differentiation and migration intrinsically in chondrocytes (Xu et al., 2021). Recently, cartilage tissue engineering (TE) has shown promise in cartilage repair *via* the improvement of biological function. Cartilage TE is a therapeutic approach that acts by orchestrating chondrocytes, scaffolds, and bioactive molecules (Li Y. et al., 2019).

TE technology is rapidly developing. A satisfactory cartilage regeneration has already been achieved *in vitro* (Xu et al., 2020c), subcutaneously in nude mice (Xu et al., 2017), and in a submuscular zone in rabbits (Xu et al., 2020b; Xu et al., 2022). Notably, successful cartilage regeneration occurs mostly when immune systems are deficient or compromised. However, when subcutaneously implanted into a large animal with immunocompetent function, tissue-engineered cartilage is exposed to inevitable severe inflammatory responses due to various reasons: 1) the scaffold itself and/or its degradation products induce inflammatory responses (Shen et al., 2019); 2) *in vitro* expanded chondrocytes present a tendency for dedifferentiation, and may be treated as allogeneic cells even in the autologous body, leading to immune rejection; 3) the trauma caused by surgical implantation can evoke inflammation (Xu et al., 2020a). These inflammatory responses deteriorate chondrocytes and degenerate the cartilage-specific extracellular matrix (ECM), which eventually leads to inferior cartilage formation. Therefore, inflammatory responses are a major obstacle to the application of cartilage TE in large animals.

Several strategies have been proposed to overcome cartilage degeneration caused by inflammatory responses. Liu et al. (2016) alleviated post-implantation inflammation in a goat model through prolonged *in vitro* pre-cultivation, which promoted the formation of a more stable subcutaneous cartilage. Li D. et al. (2019) adopted a cartilage TE technique that excluded additional materials to omit the unfavored inflammatory reactions triggered by them. Shen et al. (2019) proposed using pH-neutral fibers for developing core-shell unidirectional fibrous structures composed of chitosan/poly (lactide-co-glycolide) (CTS/PLGA). This modified polymer could neutralize the acidic products of PLGA degradation and ameliorate inflammatory responses to them. Xu et al. (2020a) reported that nanofibers could function as a physical barrier to attenuate inflammatory reactions. However, these strategies failed to show effectiveness against all causes of inflammatory responses, eventually causing unsatisfactory cartilage regeneration in a large animal model. Consequently, a more practical anti-inflammatory method is yet to be conceived.

The application of improved scaffolds with bioactive molecule controlled-releasing properties is attracting increasing attention owing to its efficacy. Parallelly, Chinese medicinal herbs have been used for treating cartilaginous diseases for several centuries and form a precious reservoir of potential remedies. One example is *Epimedium pubescens*, which has icariin (ICA, C33H40O15; molecular weight, 676.67) as its primary component. This flavonoid glucoside is a potential accelerator for cartilage TE owing to its ability to promote chondrocyte proliferation and reduce the degradation of cartilaginous ECM. ICA is a particularly safe and effective natural medicinal compound with potential anti-inflammatory properties that has been traditionally used for treating rheumatoid arthritis. Finding from a previous study indicated that ICA inhibits NF-κB expression and activates autophagy, thus suppressing inflammatory cytokines and apoptosis and eventually protecting cartilage tissues (Mi et al., 2018). ICA also alleviated inflammatory cytokine-induced ECM degradation by activating the Nrf2/ARE pathway (Zuo et al., 2019). Therefore, we conjectured that an ICA-loaded scaffold could simultaneously promote chondrogenesis and attenuate inflammatory responses in a large animal model, eventually stabilizing cartilage regeneration.

Here, we prepared ICA/CTS hydrogel by loading ICA into a routinely used chitosan (CTS) hydrogel, to endow the ICA/CTS hydrogel with concurrent chondrogenic and anti-inflammatory activities. Initially, the ICA release kinetics from the ICA/CTS hydrogel were assessed. Thereafter, the *in vitro* anti-inflammatory effects and cytocompatibility of the ICA/CTS hydrogel were evaluated. Further, the ICA/CTS hydrogel was colonized with chondrocytes and cultured *in vitro* to evaluate *in vitro* chondrogenesis. The *in vitro* engineered cartilage was then subcutaneously implanted in a large animal model (goat) to determine the feasibility of achieving stable cartilage regeneration. Finally, the anti-inflammatory effect of ICA was confirmed by examining the inflammatory response.

## Materials and methods

### Icariin/chitosan hydrogel preparation

CTS was added to an acetic acid solution (No. A6283, 0.1 M, Sigma) under mild stirring for complete dissolution. The supernatant was collected and purified by passing through 0.22-μm filters. Its pH was adjusted to 7.0 using NaOH solution (No. S5881, 1 M, Sigma). Afterward, the final concentration of the CTS solution was adjusted to 5 w/v% using centrifugal filters (3 kDa MWCO). Subsequently, β-GP (G9422, Sigma) was used as a physical crosslinker. β-GP was first dissolved in Milli-Q water to achieve 12 wt%. Then, it was added to the CTS solution to prepare a thermosensitive CTS hydrogel precursor solution.

In addition, ICA (No. I1286, purity ≥94%, Sigma) was dissolved in dimethylsulfoxide (No. 472301, Sigma) at 20 mM, and the derived solution was added to the prepared CTS (hydrogel precursor solution) till a final concentration of 10 μg/ml was achieved. Finally, a thermosensitive ICA/CTS hydrogels precursor solution was formed. Both CTS and ICA/CTS hydrogel precursor solutions were incubated at 37°C for complete gelation if necessary.

### Fourier transform infrared spectroscopic analysis

The pristine ICA and CTS, as well as the gelated ICA/CTS hydrogel, were analyzed using an ATR–FTIR (model-Alpha, Bruker, Germany) spectrometer with a scanning range from 1000 to 4,000 cm^−1^ at ambient temperature.

### Release kinetics of icariin from the icariin/chitosan hydrogel

To test the *in vitro* release of ICA from the ICA/CTS hydrogel, gelated ICA/CTS hydrogel was immersed in 10 ml of high-glucose Dulbecco’s Modified Eagle Medium (DMEM, No. D5030, Sigma) supplemented with 10% fetal bovine serum (FBS, No. 12103C, Sigma) and incubated under 5% CO_2_ at 37°C for 6 weeks. The medium was refreshed weekly. Samples were collected from the medium every week and dried. The dried samples were immersed in 10 ml of ethanol, exposed to ultrasonic vibration for 1 h, and incubated for two more days. The ICA/ethanol concentration was measured at 270 nm.

### *In vitro* degradation rate

The initial weight of the lyophilized CTS and ICA/CTS hydrogels was considered as M_0_. The gel was immersed in PBS (pH = 7.4) and incubated in an environment with 5% CO_2_ at 37°C for 6 weeks. The samples were taken out of PBS every week, dried, and weighed again as M_n_. The degradation rate was calculated as (M_0_—M_n_)/M_0_ × 100%.

### Rheological characterization

The rheological properties of the ICA/CTS and CTS hydrogels were evaluated using a controlled stress rheometer (HAAKE Rheo Stress 6000, Thermo Scientific, Germany) with parallel-plate (25 mm diameter) geometry at room temperature. The rheometer was further connected with a temperature-controlled water bath to test the dynamic storage modulus (G′) of hydrogels.

### Swelling ratio

The initial gelated CTS and ICA/CTS hydrogels were fabricated in a cubic shape (1×1×1 cm^3^), and their volume was considered to be V_0_. Thereafter, the hydrogels were immersed in PBS (pH = 7.4) and incubated in an environment with 5% CO_2_ at 37°C for 60 h. The samples were taken out of PBS every 20 h, and their volumes were measured and denoted as V_n_. The swelling ratio was calculated as follows: V_n_/V_0_ × 100%.

### *In vitro* evaluation of anti-inflammatory potential

RAW264.7 macrophages (Saimofei, China) were pre-treated with 1 μg/ml lipopolysaccharide (LPS, No. SMB00610, Sigma) for 24 h. The pre-treated RAW264.7 macrophages were inoculated into ICA alone, and ICA/CTS and CTS hydrogel precursor solutions at a density of 5.0 × 10^4^ cells/mL. Thereafter, 2 ml of macrophage-loaded hydrogel was incubated at 37°C for complete gelation and cultured in high-glucose DMEM supplemented with 5% FBS at 37°C in 5% CO_2_. After 24 h, samples from the two groups were collected for western blot (WB) analysis and real-time polymerase chain reaction (RT-PCR).

For WB examination, total protein extracts were prepared from samples using RIPA buffer (KeyGen, China). Protein samples were separated by SDS-PAGE, transferred to PVDF membranes, and blocked with 5% bovine serum albumin. Subsequently, PVDF membranes were covered with primary antibodies as probes against inflammation-related proteins, including interleukin 6 (IL-6) (1:1000, No. sc-57315, Santa Cruz Biotechnology, Inc.), tumor necrosis factor alpha (TNF-α) (1:1000, No. sc-12744, Santa Cruz Biotechnology, Inc.), and GAPDH antibodies (1:10,000, No. ab8245, Abcam). After the HRP-conjugated goat anti-rabbit/mouse IgG antibody (1:10,000, No. AS1110, Aspen Biotech, Shanghai, China) was applied, the bands formed were visualized using an ECL kit (No. AS1059-3, Aspen Biotech, Shanghai, China), and their intensity was analyzed using the Band-Scan software. The acquired images were further analyzed using the ImageJ software to quantify relative expression levels.

The expression levels of inflammation-related genes were further analyzed by RT-PCR. Total RNA extraction was performed using TRIzol™ reagent (No. 46301, Invitrogen). cDNA synthesis was performed using Moloney murine leukemia virus reverse transcriptase (No. QR0100, Invitrogen). Quantification was performed using RT-PCR with the primers (listed in Table 1) and a Fast Synergy Brands Green Master Kit and Light Cycler 480 System (Roche) according to the manufacturer’s instructions. The comparative threshold cycle method was used to analyze the results, and the expression of the endogenous reference gene *GAPDH* was used for their normalization.

**TABLE 1:** Primers used in the RT-PCR reaction.

Gene	Forward (5′-3′)	Reverse (5′-3′)
*IL-6*	GTG​GAA​GAC​AAA​CCA​TGT​TGC​CGT	TAT​TGC​AGG​TGA​GCT​GGA​CGT​TCT
*TNF-α*	AGA​ACA​GCA​ACT​CCA​GAA​CAC​CCT	TGC​CAG​TTC​CAC​ATC​TCG​GAT​CAT
*GAPDH*	CGC​TAA​CAT​CAA​ATG​GGG​TG	TTG​CTG​ACA​ATC​TTG​AGG​GAG

### Chondrocyte culture

The ethical approval required for this study was obtained from the Xuchang Central Hospital Ethics Committee. A 2–3-month-old goat was purchased from Shanghai Jiagan Experimental Animal Raising Farm (Shanghai, China). The auricular cartilage of the goat was collected and fragmented into 1 × 1 mm^2^ pieces. Following this, the cartilage was digested with 0.2% collagenase NB4 (No.17454, SERVA, Germany) for 8 h at 37°C on a shaker. Singularized chondrocytes were collected and cultured in high-glucose DMEM supplemented with 10% FBS and 1% antibiotic–antimycotic (AA). Cells were passaged at >80% confluence. Finally, cells at their second passage (P2) were harvested for further application.

### Biocompatibility evaluation

To determine the biocompatibility of ICA/CTS and CTS hydrogels, chondrocytes were evenly mixed with ICA, and the ICA/CTS and CTS precursor solutions. The final concentration was adjusted to 5.0 × 10^5^ cells/mL. This cell concentration was suitable for complete gelation after temperature enhancement to 37°C. Thereafter, the chondrocyte-loaded hydrogels were cultured *in vitro* in DMEM supplemented with 10% FBS/1% AA for 7 days. After 1, 4, and 7 days, the cell viability in the hydrogels was measured using a live/dead cell viability assay (No. 34862, Invitrogen) and measured using a confocal microscope (Nikon, Japan). ImageJ was used to quantitatively analyze the number of live cells from the acquired images. The cell proliferation levels in the samples were evaluated by measuring the absorbance of a cell counting kit-8 reagent (CCK-8, No. C0040, Beyotime, Shanghai, China) at 450 nm.

### *In vitro* chondrogenesis

Chondrocytes were homogenously mixed within ICA/CTS and CTS hydrogel precursor solutions (1 × 10^7^ cells/mL) and gelated at 37°C. The cell suspensions were cultured in DMEM supplemented with 10% FBS/1% AA at 37°C in 5% CO_2_. After 2 weeks of cultivation, samples from the ICA/CTS and CTS groups were retrieved for histological and biochemical evaluations.

For histological assessment, the specimens were fixed, decalcified, paraffin-embedded, and cut into sections of 5 μm thickness. Samples were stained with hematoxylin-eosin (HE) for morphological evaluation. Safranin-O staining was used for assessing glycosaminoglycan (GAG) distribution. Finally, the cartilage-specific ECM was analyzed by immunohistochemical staining for collagen II.

In addition, cartilage-related biochemical components of the retrieved samples from the ICA/CTS and CTS groups were further estimated. Samples (*n* = 3) were collected and minced to quantify DNA, GAG, and type II collagen using the PicoGreen dsDNA assay (No. 10225ES84, Invitrogen), dimethyl methylene blue assay (Sigma), and Collagen II Detection Kit (No. 6009, Chondrex Inc. Redmond, WA, United States), respectively. The DNA, GAG, and collagen II contents were normalized to the tissue wet weight. In addition, GAG/DNA and collagen II/DNA data were also calculated.

### Cartilage regeneration subcutaneously implanted in a goat

After 2 weeks, the *in vitro* engineered cartilage from both ICA/CTS and CTS groups was subcutaneously implanted into the dorsal area of the autologous goat. At three- and 6-week post-implantation, samples were harvested for cartilage-related evaluation and inflammatory assessment.

For cartilage evaluation, samples were histologically stained using HE, Safranin-O, and immunohistochemical collagen II reagents. Additionally, GAG and collagen II were further evaluated as cartilage-related biochemical components, in which the GAG and collagen II contents were normalized to the tissue wet weight. Briefly, total GAG was precipitated using guanidinium chloride solution (0.98 mol/L). The OD values were determined at 595 nm after the GAG precipitate was dissolved. A standard curve was prepared using chondroitin-4-sulfate, and the total GAG content was measured from the OD value correlating to the corresponding GAG content in the standard curve. The total collagen content was quantified in the hydroxyproline assay. Samples were prepared by alkaline hydrolysis, and free hydroxyproline hydrolyzates were assayed. The hydroxyproline content was converted to the total collagen content according to a collagen to hydroxyproline mass ratio of 7.25.

The expression levels of the inflammatory cytokines interleukin 1β (IL-1β), IL-6, and TNF-α and an apoptosis-related marker (TUNEL) in the samples were analyzed by immunofluorescence staining. Samples were treated overnight with primary antibodies against collagen type II, IL-1β, IL-6, TNF-α, and TUNEL (No. ab188570, ab254360, ab303458, ab183218, and ab66108, Abcam) at 4°C. After rinsing, the samples were treated with goat anti-rabbit secondary antibody (1:1000, ab150077, Abcam) in dark. Finally, coverslips were mounted with 4′,6-diamidino-2-phenylindole (No. 10236276001, DAPI, Sigma) mounting solution, visualized using a fluorescent imaging system, and analyzed by a fluorescence microscope (Olympus). The acquired images were analyzed with ImageJ software to calculated the relative intensity as follows: M/N × 100%, where M is the occupied area of the positive expressed marker and N is the area of the total image.

### Statistical analysis

Data were reported as mean value ±standard deviation (SD) and analyzed using SPSS version 17.0 software. Inter-group differences were analyzed by Student’s t-test. All experiments were performed in triplicate. *p* < 0.05 denoted a statistically significant difference.

## Results

### Fabrication of icariin/chitosan hydrogel

**FIGURE 1:**
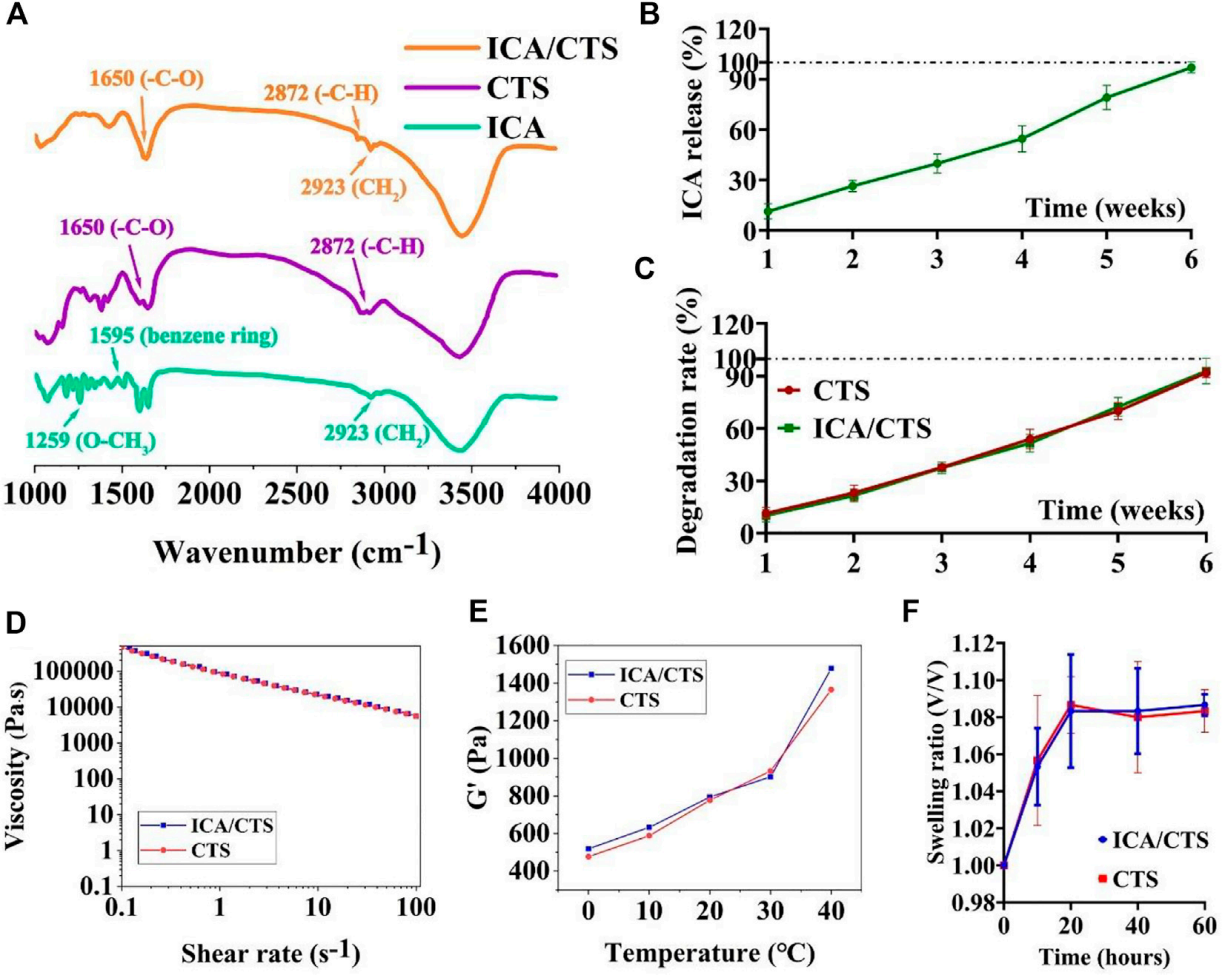
FTIR, ICA release kinetics, and degradation rate examinations. FTIR examination of pristine ICA and CTS, as well as ICA/CTS samples **(A)**. *In vitro* ICA release kinetics in ICA/CTS hydrogel immersed in PBS for 6 weeks **(B)**. *In vitro* degradation rate of CTS and ICA/CTS hydrogels immersed in chondrocyte co-culture medium for 6 weeks **(C)**. Viscosity **(D)**, dynamic storage moduli (G′) **(E)**, and swelling ratio **(F)** of CTS and ICA/CTS hydrogels. (*n* = 3; data are presented as means ± SD for each group).

To endow the CTS hydrogel with concurrent chondrogenesis and anti-inflammatory effect, ICA was loaded into the CTS hydrogel to prepare a composite ICA/CTS hydrogel. FTIR spectral data were used to confirm the chemical stability of ICA in the ICA/CTS hydrogel (Figure 1A). For pristine CTS samples, the characteristic peaks at 2872 and 1650 cm^−1^ were attributed to the -C-H and carbonyl (-C-O) stretching vibrations of the secondary amide (amide I band), respectively. The characteristic peaks of ICA appeared at 2923, 1595, and 1259 cm^−1^, corresponding to methylene (CH_2_), benzene ring, and methoxyl (O–CH_3_), respectively. Both the characteristic peaks of pristine CTS and ICA samples were observed in the ICA/CTS sample, indicating the successful preparation of the ICA/CTS hydrogel. Notably, the characteristic peak of ICA at 2921 cm^−1^ appeared in the ICA/CTS hydrogel with no change, suggesting zero chemical change in ICA during ICA/CTS hydrogel production, which could have helped retain the original bioactivity of ICA.

We further conducted *in vitro* experiment to illustrate the release kinetics of ICA from the ICA/CTS hydrogel. Our data indicated that ICA presents sustained release kinetics from the ICA/CTS hydrogel when immersed in chondrocyte co-culture medium. Almost 100% percent of ICA was completely released from the ICA/CTS hydrogel at 6 weeks (Figure 1B). In addition, our data revealed that the degradation rate of CTS and ICA/CTS hydrogels exhibited a similar trend with the release kinetics of ICA (Figure 1C), suggesting that the addition of ICA did not affect the rate of CTS hydrogel degradation. Thus, the release kinetics of ICA could be governed by the degradation of CTS.

In addition, both ICA/CTS and CTS hydrogels exhibited similar shear-thinning, storage modulus (G′), and swelling ratio behaviors (Figures 1D–F), suggesting that the addition of ICA did not affect the rheological, gelation, and swelling properties of the CTS hydrogel. These results indicated that the ICA/CTS hydrogel retained the properties of original CTS hydrogel for use in cartilage regeneration.

### *In vitro* anti-inflammatory effect of icariin/chitosan hydrogel

**FIGURE 2:**
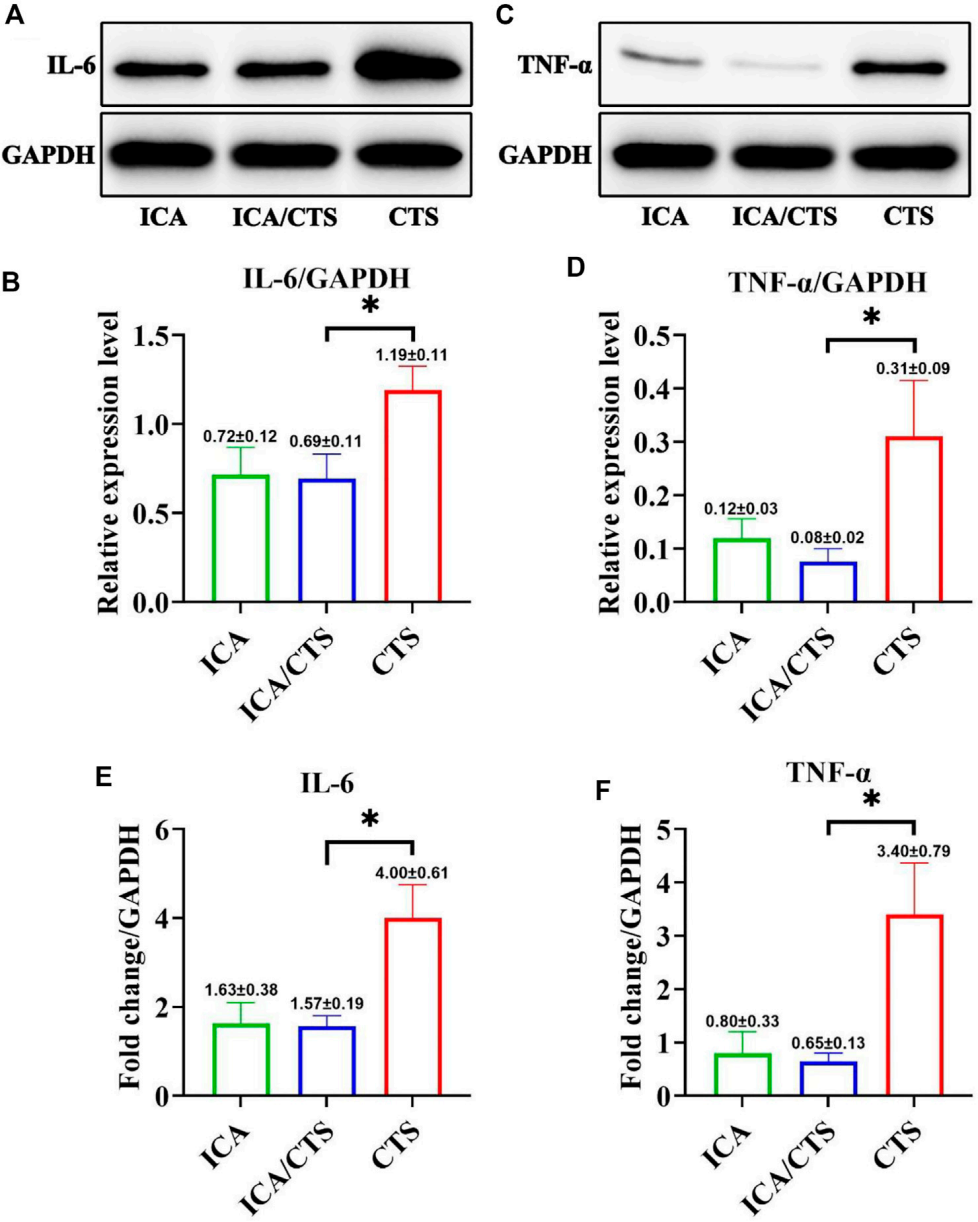
Evaluation of *in vitro* anti-inflammatory effects of ICA/CTS and CTS hydrogels incubated with RAW264.7 macrophages for 24 h. The protein expression levels of IL-6 **(A,B)** and TNF-α **(C,D)** detected by WB examination in ICA/CTS and CTS groups. Gene expression of IL-6 and TNF-α detected by RT-PCR in the ICA/CTS and CTS groups **(E,F)**. (*n* = 3; data are presented as means ± SD for each group, *p < 0.05).

To test the anti-inflammatory properties of the ICA/CTS hydrogel, RAW264.7 macrophages were pre-induced with LPS and incubated with ICA, ICA/CTS, and CTS hydrogels for 24 h. WB examination indicated a significant attenuation in the protein expression of all inflammatory cytokines (including IL-6 and TNF-α) in the ICA/CTS group compared to that in the CTS group (Figures 2A–D). RT-PCR examination further confirmed that all inflammatory protein-encoding genes (including IL-6 and TNF-α) were expressed at lower levels in the ICA/CTS group than in the CTS group (Figures 2E,F). Thus, the addition of ICA endowed the ICA/CTS hydrogel with pronounced anti-inflammatory properties. In addition, our data revealed that the expressions of both IL-6 and TNF-α in the ICA group was similar to that in the ICA/CTS group, suggesting that CTS fabrication does not affect the anti-inflammatory potential of ICA.

### Cytocompatibility of icariin/chitosan hydrogel

**FIGURE 3:**
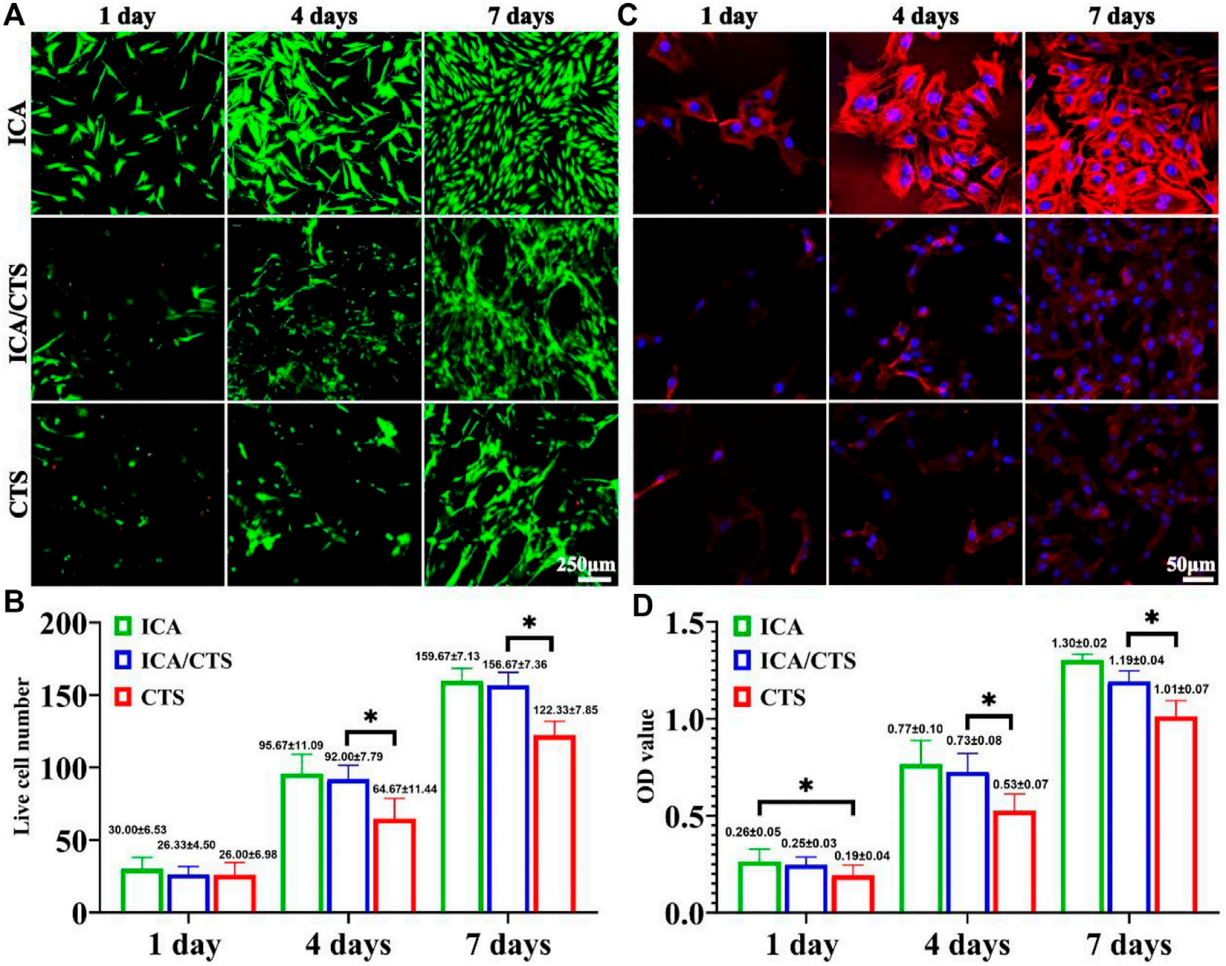
Cytocompatibility evaluation of ICA/CTS and CTS hydrogels with *in vitro* co-cultured with chondrocytes for 1–7 days. Live/dead staining of samples in the ICA, ICA/CTS, and CTS groups **(A)**. Quantitative analysis of live cell number **(B)**. Phalloidin staining images in ICA, ICA/CTS, and CTS groups **(C)**. OD value detected in the CCK-8 assay in ICA, ICA/CTS, and CTS groups **(D)**. (*n* = 3; data are presented as means ± SD for each group, *p < 0.05).

The cytocompatibility of ICA/CTS was evaluated *in vitro* by co-culture with chondrocytes for 7 days. Live/dead staining revealed an increasing trend of cell number with increased incubation time from 1 to 7 days in the ICA, ICA/CTS, and CTS groups (Figures 3A,B). Scarcely dead cells were observed in both groups, indicated by the negligible number of red-stained cells. Notably, cell number were greater in the ICA and ICA/CTS groups than in the CTS group. In addition, phalloidin staining revealed that the ICA group had greater F-actin expression and stretching than both ICA/CTS and CTS groups, suggesting that the chondrocytes changed their morphology in response to hydrogel treatment (c). Consistent with the above results, the OD values determined from the CCK-8 assay were elevated with the increase in culture duration, and the OD values in the ICA and ICA/CTS groups were greater than those in the CTS hydrogel (Figure 3D). The results indicated that both the ICA/CTS and CTS hydrogels were highly cytocompatible, and that the addition of ICA significantly promoted chondrocyte proliferation.

### *In vitro chondrogenesis* of the icariin/chitosan hydrogel

**FIGURE 4:**
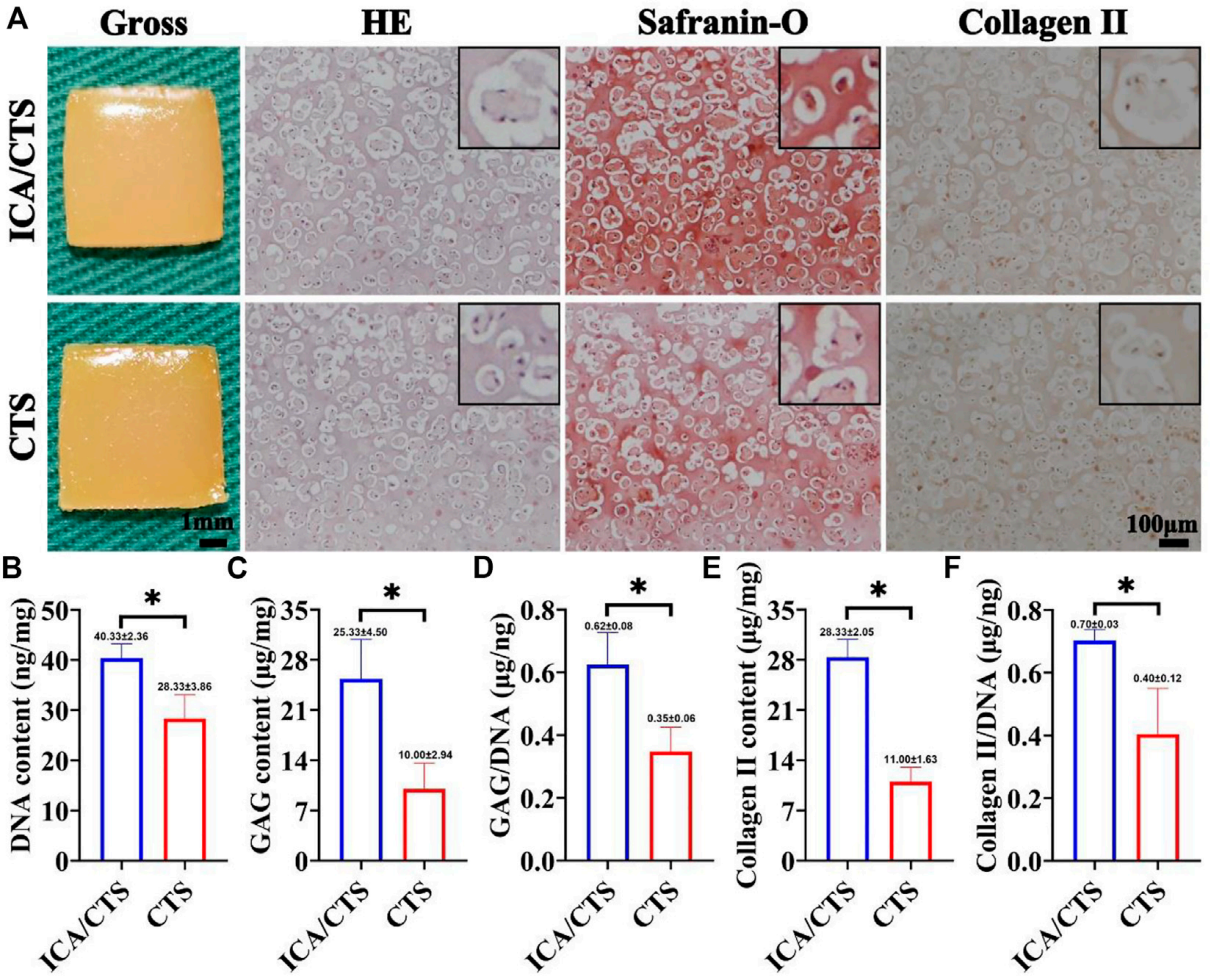
*In vitro* chondrogenesis in ICA/CTS and CTS hydrogels colonized with chondrocytes for 2 weeks. Gross, HE, Safranin-O, and collagen type II immunohistochemical staining of samples in ICA/CTS and CTS groups **(A)**. The black-framed images represent the magnified views. Quantitative analyses of DNA, GAG, GAG/DNA, collagen type II contents, and collagen II/DNA in ICA/CTS and CTS groups **(B–F)**. DNA, GAG, and collagen II normalized to tissue wet weight. (*n* = 3; data are presented as means ± SD for each group, *p < 0.05).

Both ICA/CTS and CTS hydrogels colonized with chondrocytes and were subjected to culture for 2 weeks to verify the *in vitro* chondrogenesis potential. According to our positive results from safranin-O and immunohistochemical collagen II staining methods, samples in both ICA/CTS and CTS hydrogels displayed apparent cartilage-specific ECM deposition (Figure 4A). Of note, both safranin-O and collagen II staining test results indicated more intensive staining in ICA/CTS hydrogel samples than in CTS hydrogel samples. In addition, the quantitative analyses of cartilage-related biochemical components, including DNA, GAG, GAG/DNA, collagen II, and collagen II/DNA, showed an obvious increasing trend in the ICA/CTS hydrogel than in the CTS hydrogel (Figures 4B–F). Notably, the higher GAG/DNA and collagen II/DNA levels in the ICA/CTS group than in the CTS group suggested that the ICA/CTS hydrogel contributed to greater chondrogenic ECM production but not owing to a larger chondrocyte population. The results suggested that both ICA/CTS and CTS hydrogels can be used for cartilage regeneration *in vitro* and ICA addition can promote significant chondrogenesis.

### Stable cartilage regeneration using icariin/chitosan hydrogel in a goat model

**FIGURE 5:**
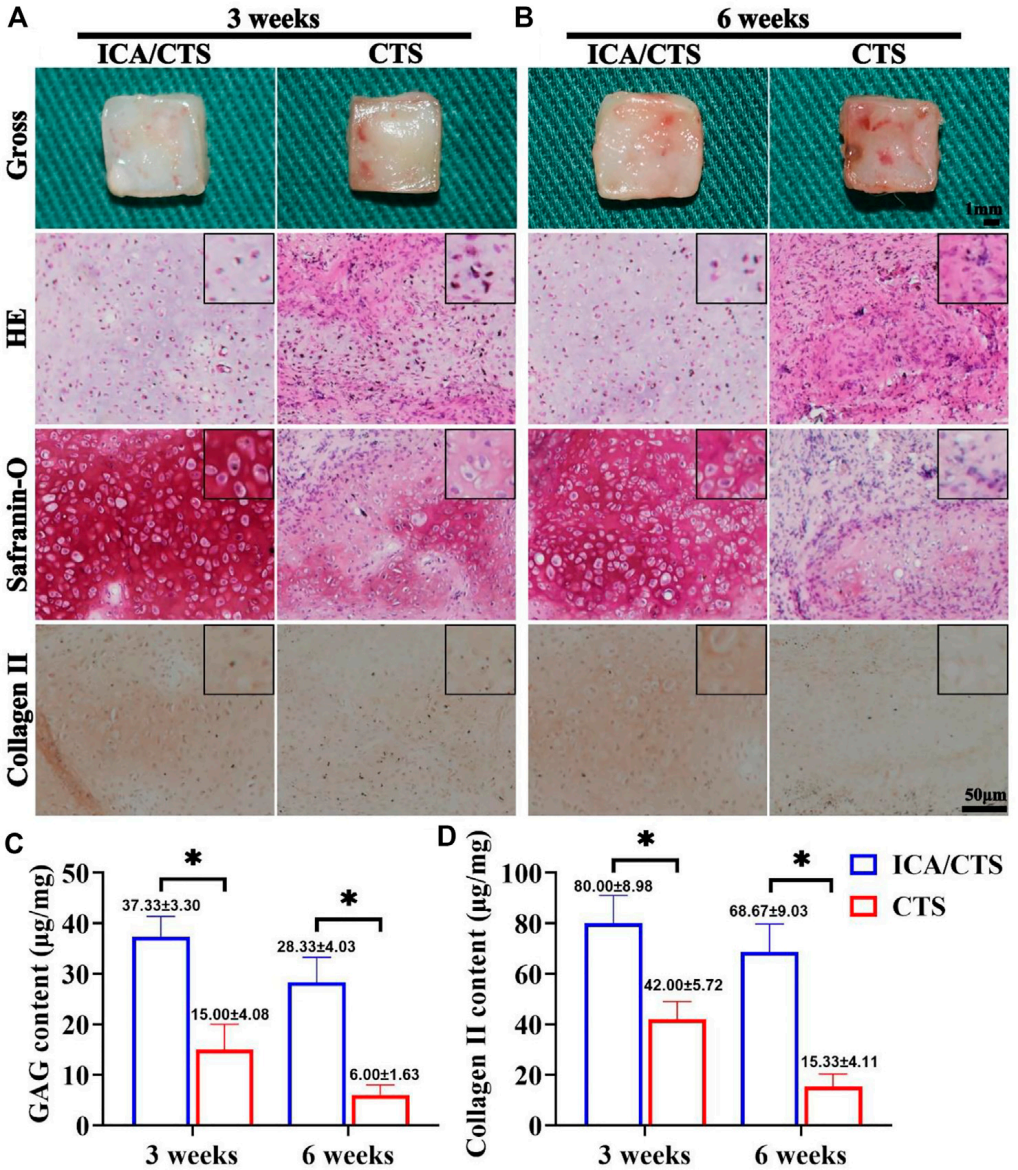
Cartilage regeneration of samples in the ICA/CTS and CTS groups after subcutaneous implantation in goats for 3 and 6 weeks. Gross, HE, Safranin-O, and collagen type II immunohistochemical staining of samples in the ICA/CTS and CTS groups at 3 weeks **(A)** and 6 weeks **(B)**. The black-framed images represent the magnified views. Quantitative analyses of GAG and collagen type II contents in the ICA/CTS and CTS groups **(C,D)**. GAG and collagen II contents normalized to the tissue wet weight. (*n* = 3; data are presented as means ± SD for each group, *p < 0.05).

The *in vitro* regenerated cartilage samples from both ICA/CTS and CTS hydrogels were subcutaneously implanted in an autologous goat model and subject to *in vivo* incubation for 3 and 6 weeks to confirm the feasibility of cartilage formation in an immunocompetent large animal. Compared to CTS hydrogels, ICA/CTS hydrogel samples had more markable cartilage-specific ECM secretion and a typical lacuna-like structure at both weeks three and six post-implantation. This result was confirmed by both HE staining and the more intense safranin-O and collagen II staining methods (Figures 5A,B). Additionally, cartilage-specific ECM showed a declining trend, and a typical lacuna structure was observed for a long duration in samples from both ICA/CTS and CTS hydrogels. The above results were further confirmed by the quantitative analysis results of cartilage-related biochemical components. In these analyses, higher levels of GAG and collagen II were observed in the ICA/CTS group than in the CTS group, and lower levels were observed after a considerable duration (Figures 5C,D). The results indicated that the ICA/CTS hydrogel exhibited potential for subcutaneous cartilage regeneration in a goat, and the addition of ICA significantly promoted cartilage regeneration in an immunocompetent large animal model.

### Inflammatory reaction evaluation

**FIGURE 6:**
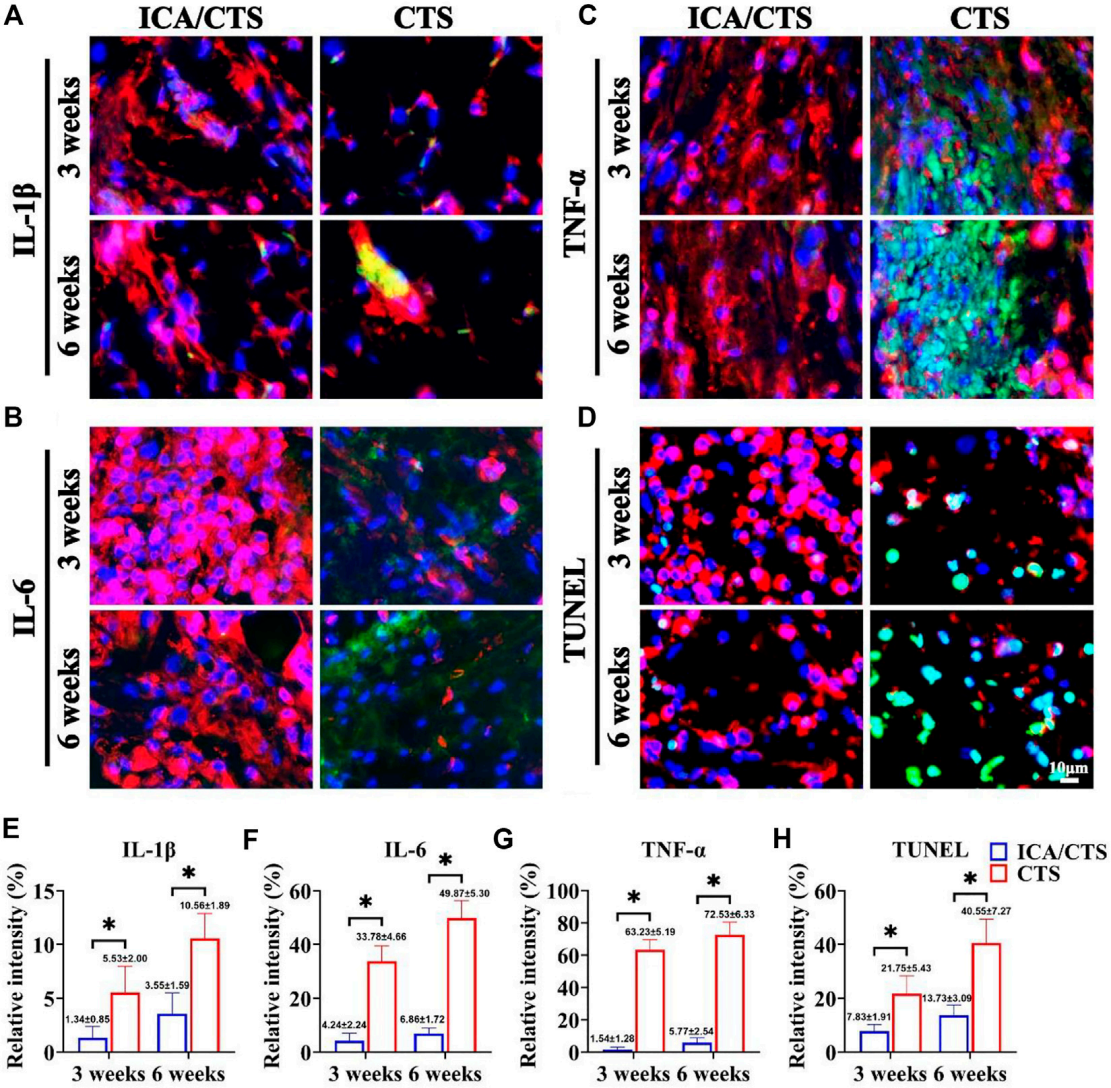
Inflammatory reaction of samples in the ICA/CTS and CTS groups after subcutaneous implantation in goats for 3 and 6 weeks. Immunofluorescence staining for IL-1β, IL-6, TNF-α, and TUNEL in samples from the ICA/CTS and CTS groups [**(A–D)**, marked with green color], in which a chondrogenic marker of collagen type II was stained with red color. Relative intensity corresponding to the positive expression of IL-1β, IL-6, TNF-α, and TUNEL in the ICA/CTS and CTS groups **(E–H)**. (*n* = 3; data are presented as means ± SD for each group, *p < 0.05).

The cartilage tissue generated from ICA/CTS and CTS hydrogels was evaluated for inflammatory reactions to elucidate the mechanism of action of ICA in promoting cartilage regeneration in an immunocompetent large animal. More intensive immunofluorescence staining of all inflammation-related factors (IL-1β, IL-6, and TNF-α) and apoptosis-related marker (TUNEL) was observed with time over 3–6 weeks in both ICA/CTS and CTS hydrogels (Figures 6A–D). The immunofluorescence of IL-1β, IL-6, TNF-α, and TUNEL was attenuated in the ICA/CTS hydrogel compared to that in CTS hydrogel. Quantitative analyses further confirmed that the relative intensity of IL-1β, IL-6, TNF-α, and TUNEL was elevated with time over 3–6 weeks, and lower levels were observed in ICA/CTS hydrogels than in the CTS hydrogel (Figures 6E–H). Collectively, these results demonstrated that the addition of ICA endowed the ICA/CTS hydrogel with a pronounced anti-inflammatory property.

## Discussion

To date, stable cartilage regeneration in an immunocompetent large animal remains challenging, owing to the absence of effective drugs and methods that concurrently promote chondrogenesis and prevent the degeneration of engineered cartilage caused by inflammatory erosion. In this study, ICA, a potent chondrogenic accelerator and anti-inflammatory agent, was loaded into CTS hydrogel to develop an effective platform for promoting cartilage regeneration in a large animal. Our findings demonstrated that the ICA/CTS hydrogel steadily releases ICA to simultaneously exert chondrogenic and anti-inflammatory effects. The ICA/CTS hydrogel was cytocompatible and promoted chondrocyte proliferation and cartilage-specific ECM secretion *in vitro* when colonized with chondrocytes. Notably, satisfactory cartilage regeneration was achieved when chondrocyte-loaded ICA/CTS hydrogels were subcutaneously implanted in a goat model, primarily owing to the concurrent chondrogenic and anti-inflammatory effects of ICA. The findings of this study suggested that ICA is an effective candidate for cartilage regeneration and treating cartilage damage in a large animal.

Extensive evidence suggests that growth factors, including transforming growth factor beta (TGF-β), bone morphogenetic proteins (BMPs), and insulin-like growth factor (IGF) can stimulate chondrogenic differentiation, promote chondrocyte proliferation, and enhance cartilage generation. These growth factors facilitate the deposition of cartilage-specific ECM components, including collagen type II. However, they inevitably cause some side effects, such as hypertrophic differentiation and fibrous ECM formation. Furthermore, other drawbacks related to growth factors, including high price, rapid degradation, and short activity time, significantly limit their widespread application. These growth factors particularly lack anti-inflammatory potential, which leads to failure in protecting the engineered cartilage from inflammatory erosion and causes unstable cartilage regeneration in an immunocompetent large animal. Therefore, developing safe and low-cost drugs that promote chondrogenesis and exert anti-inflammatory activities in parallel is an urgent need.

In recent times, traditional Chinese medicine has gained attention for its availability, affordability, and consistent efficacy. In the same regard, the potential of active ingredients used in Chinese medicine for protecting and repairing cartilage tissues has garnered the interest of researchers (Yang et al., 2018). ICA flavonoids are the primary component of *Epimedium* and have multiple pharmacological properties. Recently, they have been considered as accelerants that affect cartilage tissue (Zhang et al., 2020). Also, they are been used increasingly in tissue engineering applications to promote chondrogenic differentiation in cartilage tissues and reduce hypertrophic differentiation (Wang et al., 2014). ICA regulates the chondrocyte’ autophagy *via* the PI3K/AKT/mTOR signaling pathway and preserves the cartilage phenotype (Tang et al., 2021). Besides, ICA is an inexpensive non-immunogenic agent with consistent efficacy compared to exogenous growth factors (Zhang et al., 2012). Furthermore, ICA is known to preserve the phenotype of chondrocytes and facilitate their proliferation. Consistent with these findings, our data also confirmed that the released ICA from ICA/CTS hydrogel significantly promotes chondrocyte proliferation, as indicated by results from live/dead staining, CCK-8 assay, and DNA content evaluation.

Findings from several studies suggest a chondrogenesis-promoting effect of ICA not only in chondrocytes but also in mesenchymal stem cells, mediated by the secretion of various chondrogenic growth factors, such as BMPs and TGF-β1 (Kankala et al., 2018; Tang et al., 2022). Literature evidence suggests that BMP signals can significantly upregulate the expression of a key chondrogenic gene (Sox9), collagen type II, and aggrecan (Li et al., 2012; Lin et al., 2020). Data from this study confirmed that the ICA released from ICA/CTS hydrogels enhanced the secretion of cartilage-specific ECM components, including both GAG and collagen II.

Considering that cartilage tissue is composed of chondrocytes surrounded by a dense cartilage-specific ECM, to promote cartilage regeneration subcutaneously in an immunocompetent large animal, an ideal drug should simultaneously promote chondrocyte proliferation and suppress cartilage ECM degeneration. Findings from some reports confirmed that ICA could suppress nitric oxide (NO) and matrix metalloproteinase (MMP) synthesis, eventually inhibiting cartilage-specific ECM degradation (Liu et al., 2010). Therefore, ICA can be considered a safe and potential chondrocyte anabolic drug with protective effects against ECM destruction probably through NO and MMP synthesis inhibition. In turn, the preserved ECM regulates chondrocyte proliferation and differentiation for maintaining homeostasis and the regeneration of cartilage tissue.

Stable cartilage regeneration poses a tremendous obstacle in immunocompetent large animals for researchers worldwide. The key reason is the inevitable triggering of an inflammatory reaction of the tissue-engineered cartilage when it is subcutaneously implanted in an immunocompetent large animal model (Xia et al., 2019). Also, the catabolic activities of inflammatory cytokines, including IL-1β, IL-6, and TNF-α, cause cartilage ECM degradation by promoting the expression of MMP and other enzymes, such as a disintegrin and metalloproteinase with thrombospondin motifs (ADAMTS) (Chen et al., 2015). Another study reported a regulatory effect of IL-1β and TNF-α on chondrogenic genes such as SOX9, collagen type II, and aggrecan (reducing effect) as well as MMP (increasing effect) (Ouyang et al., 2019). TNF-α is also involved in cartilage degeneration, which has been shown to activate NF-κB and PI3K/AKT (Li W. et al., 2019).

Hence, the development of a platform loaded with anti-inflammatory drugs is a reasonable avenue for reversing the degeneration of cartilage ECM in immunocompetent large animals. A previous study demonstrated that ICA exhibits both antioxidative (Sze et al., 2010) and anti-inflammatory (Xu et al., 2010) properties. ICA has also been shown to protect chondrocytes from inflammatory responses during septic arthritis (Liu et al., 2010) and promote ECM synthesis (Zhang et al., 2012) by neutralizing IKK and IκB phosphorylation. This promotes the suppression of NF-κB/HIF-2α signaling pathways (Wang et al., 2020a). Another anti-inflammatory function of ICA is alleviating LPS-induced acute immune responses, such as pyroptosis, which is reportedly mediated by the inhibition of NLRP3 inflammasome-mediated caspase-1 signaling (Zu et al., 2019), activation of PI3K/Akt pathway, and inhibition of NF-κB (2013). Liu et al. (2010) reported the same protective effect of ICA against LPS-induced inflammation in chondrocytes. In this study, our *in vitro* data confirmed that ICA released from the ICA/CTS hydrogel could downregulate IL-6 and TNF-α as the primary cytokines acting against LPS-induced inflammatory. In addition, findings from our *in vivo* study further revealed that ICA released from the ICA/CTS hydrogel could promote cartilage tissue regeneration in the large immunocompetent animal model by attenuating the inflammatory response, as evidenced by the attenuated expression of IL-1β, IL-6, and TNF-α. A previous study also confirmed the inhibitory effect of ICA on the expression of oxygen-glucose deprivation-induced inflammatory factors (IL-1β, IL-6, and TNF-α) through the IRE1/XBP1 pathway in microglial cells (Mo et al., 2021).

The pathological properties of cartilage degeneration include chondrocyte apoptosis and ECM synthesis reduction (Wang et al., 2020a). Nevertheless, the high rate of apoptosis in chondrocytes restricts the benefits obtained from the treatment effects on cartilage defects in long-term applications. To overcome this limitation for chondrocyte application in cartilage regeneration and cartilage defect repair, developing an effective strategy for prolonging chondrocyte vitality is an important issue. In the same regard, ICA can be used for increasing chondrocyte’ survival because it inhibits inflammation-induced injuries, probably by inhibiting the NF-κB/HIF-2α signaling pathway and promoting hypoxia-inducible factor-1α (HIF-1α) expression and anaerobic glycolysis (Wang et al., 2020b). Another study reported that ICA protected rabbit bone marrow stromal cells (BMSCs) from apoptosis induced by oxygen, glucose, and serum deprivation by inhibiting endoplasmic reticulum stress-mediated autophagy *via* the MAPK signaling pathway (Liu et al., 2020). The findings of the current study indicate that ICA can protect chondrocytes from apoptosis, as indicated by attenuated TUNEL protein expression.

CTS is a commonly-used hydrogel for cartilage regeneration as it can be easily loaded with cells, growth factors, and drugs (Oryan and Sahvieh, 2017). Our results confirmed that ICA was suitable for loading into CTS hydrogels without chemical change, which could help preserve the original bioactivity of ICA. In addition, our results indicated that ICA was released in a sustained manner and the degradation rate of CTS and ICA/CTS hydrogels showed a very similar trend with the release kinetics of ICA, suggesting that the release kinetics could be governed by the degradation of CTS. There are at least three advantages of ICA/CTS over ICA alone in the regeneration of cartilage: 1) ICA is unsuitable for rapid degradation and imparts short-term protection to regenerated cartilage. In contrast, the encapsulation of ICA into CTS prolongs the degradation course of ICA remarkably, with the ICA/CTS hydrogel showing a sustained release effect and enhancing cartilage regeneration considerably; 2) the encapsulation of ICA into CTS facilitates the localized release of ICA, resulting in precise protection to regenerated cartilages; 3) the utilization of CTS hydrogels is convenient for the shape control of regenerated cartilage.

Admittedly, the current study had several limitations: 1) gradual cartilage ECM erosion was observed over the course of treatment in subcutaneous implantation in the goat, suggesting that the optimal concentration of ICA loaded in CTS requires further investigation; 2) more sophisticated strategies should be developed to load ICA with spatiotemporal controllability and substantially prolong the biological half-life of ICA; 3) the duration for which the ICA/CTS hydrogel-generated cartilage could maintain stability in a goat model should be determined.

## Conclusion

In the current study, we successfully prepared an ICA/CTS hydrogel with both chondrogenic and anti-inflammatory properties. ICA showed sustained release from the ICA/CTS hydrogel and was biologically active to exhibit inflammatory activity both *in vitro* and *in vivo*. Additionally, the ICA/CTS hydrogel was biocompatible and significantly enhanced chondrocyte proliferation and cartilage-specific ECM deposition *in vitro*. More importantly, the chondrocyte-loaded ICA/CTS hydrogel achieved satisfactory cartilage regeneration when subcutaneously implanted in a goat model. The current study provided a reliable ICA-loading strategy that can be used to simultaneously impart chondrogenic and anti-inflammatory properties, resulting in the stable regeneration of cartilage tissue in a large immunocompetent animal model.

## Data Availability

The original contributions presented in the study are included in the article/Supplementary Material, further inquiries can be directed to the corresponding author.
